# Water-Soluble Polyglycidol-Grafted Ladder Calix Resorcinarene Oligomers with Open Chain and Cyclic Topologies: Synthesis, Characteristics, and Biological Evaluation

**DOI:** 10.3390/polym16223219

**Published:** 2024-11-20

**Authors:** Hristo Penchev, Erik Dimitrov, Christo Novakov, Emi Haladjova, Ralitsa Veleva, Veselina Moskova-Doumanova, Tanya Topouzova-Hristova, Stanislav Rangelov

**Affiliations:** 1 Institute of Polymers, Bulgarian Academy of Sciences, Akad. G. Bonchev St., Block 103A, 1113 Sofia, Bulgaria; e_dimitrov@polymer.bas.bg (E.D.); hnovakov@polymer.bas.bg (C.N.); ehaladjova@polymer.bas.bg (E.H.); 2 Centre of Competence “Sustainable Utilization of Bio-Resources and Waste of Medicinal and Aromatic Plants for Innovative Bioactive Products” (CoC BioResources), 1000 Sofia, Bulgaria; ralitsa_veleva@biofac.uni-sofia.bg (R.V.); moskova@biofac.uni-sofia.bg (V.M.-D.); topouzova@biofac.uni-sofia.bg (T.T.-H.); 3 Department of Cellular and Developmental Biology, Faculty of Biology, Sofia University St Kliment Ohridski, 8 Dragan Tzankov Blv, 1164 Sofia, Bulgaria

**Keywords:** ladder oligomers, cyclic oligomers, calix-resorcinarene, Noria, polyglycidol, MALDI-TOF, analytical ultracentrifugation, static light scattering, cytotoxicity, apoptosis

## Abstract

Ladder oligomers containing calixarene skeletons in the main chain—calix[4]resorcinarene (CRA) ladder macromolecules with open chain and cyclic macromolecules with double ring-like (Noria-type) topologies—bring particular research attention as functional materials with various applications. However, there is still a remarkable lack of studies into the synthesis of fully water-soluble derivatives of these interesting macromolecules. Research on this topic would allow their bio-based research and application niche to be at least revealed. In the present study, a strategy for the synthesis of water-soluble polyglycidol-derivatized calix resorcinarene ladder oligomers with open chain and cyclic structures is introduced. A *grafting from* approach was used to build branched or linear polyglycidol chains from the ladder scaffolds. The novel structures were synthesized in quantitative yields and fully characterized by NMR, FTIR and UV–vis spectroscopy, gel permeation chromatography, MALDI-TOF mass spectrometry, analytical ultracentrifugation, and static light scattering to obtain the molar mass characteristics and composition. The biocompatibility and toxicity of the two polyglycidol-derivatized oligomers were investigated and the concentration dependence of the survival of three cell lines of human origin determined. The selective apoptosis effect at relatively low dissolve concentrations toward two kinds of cancerous cell lines was found.

## 1. Introduction

Natural and synthetic polymers with macrocyclic ring-like architectures and topologies could be considered as a special class of macromolecules within the other three major distinguished categories: linear, cross-linked, and branched polymers [1]. Obtained and described as early as the 1940s, cyclic resorcinol-based and para-substituted phenol-formaldehyde “condensates”, later given the name “calixarenes” [2,3,4] still reveal peculiar findings in macrocyclic compounds research and application. During the last three decades, this class of macrocyclic molecules, serving as cavitands, stand as a key skeleton in the emerging host–guest chemistry development, and they find diverse applications such as optic sensors, and in specific ion detection applications [5,6] as catalysts [7], in lithography and microelectronics [8,9,10,11,12,13].

In his pioneering work, Pietro Maravigna reported the reaction products of bisphenol A, hydroquinone, or dihydroxynaphthalenes with the shortest skeleton glyoxal dialdehyde and methanesulfonic acid as a condensing agent, leading to polymeric materials having linear ladder structures and possessing a high thermal stability [14]. In 2006, by using a similar approach to that employed for the synthesis of calix/resorcin[n]arenes, namely dynamic covalent chemistry (DCC) conditions, Hiroto Kudo et al. showed that the condensation reaction of resorcinol with 50 wt.% aq. 1,5-pentanedial (glutaraldehyde) gives a unique double-cyclic ladder-type oligomer with a central cavity that structurally resembles a waterwheel, and the compound was Latin-named as Noria [15]. The same team tried a similar addition–condensation reaction of resorcinol and 1,4-butanedial and claimed to have obtained calix[4]resorcinarene (CRA) ladder polymer with four repeating CRA motifs and an open (not cyclic) chain structure [16]. Rahmatpour et al. used the more stable 2,5-dimethoxytetrahydrofuran aldehyde precursor and several dihydroxysubstituted aromatic compounds resulting in structurally similar calixarene type ladder polymers [17]. Nowadays, the research into the peculiar macrocyclic Noria and CRA-type ladder polymers gradually intensifies by employing a wide range of aliphatic dialdehydes, resulting in the synthesis and characterization of a whole library of macromolecules with different structures and topologies [18]. Further research has been carried out on the chemical modification and synthesis of Noria-type conjugates, such as esters, partially ethoxylated or methoxylated or quaternary-ammonium derivatized Noria macrocycles with intended possible application in lithography, as hosts for chemically and thermally robust Type II porous liquids for methane hosting, for supramolecules impregnated proton electrolyte membranes, and as an ionomeric cross-linking agent [19,20,21,22]. Despite the enormous potential of dialdehyde-resorcin[n]arene macrocyclic compounds, prepared by relatively easy and affordable synthetic pathways, to serve as bioactive host–guest cavitands or by its own polyphenolic bioactivity and antioxidant properties, until now and from our knowledge, there is no such literature report. One of the possible explanations of this fact is that the Noria and CRA ladder oligomers share the distinction of being water insoluble at acidic and neutral/physiological pH, which is attributed to the isoelectric point of the acidic resorcinate OH groups (pKa = 9.15 [23]), forming intermolecular H-bond networks and hydrophobic aliphatic arm bridges. The deprotonation of these groups by alkalization, for example, by treatment with diluted sodium or potassium hydroxide solutions, imparts water solubility but the need of highly alkaline media (pH above 9) and the susceptibility to oxidation of the resorcinate structure limits the bio-based application of the alkali salts of the oligomers. Therefore, functionalization with polar groups and the attachment of hydrophilic polymer chains are considered as potential synthetic approaches to addressing this issue. As far as the latter is concerned, the polymer of choice seems to be poly(ethylene glycol) (PEG). Indeed, PEGylation—a strategy of attachment or conjugation of PEG to, e.g., proteins, peptides, drugs, antibodies, any other hydrophobic molecules, nano- and microparticles or even macrostructures—has been repeatedly shown to provide solubility and stability of those species, to increase blood circulation half-life, and to impart *Stealth* properties [24,25,26,27,28,29]. PEG, however, is not free of shortcomings related to the lack of functional groups, possible immunogenicity, and potential safety issues such as antibody formation, vacuolation, and hypersensitivity [30,31,32], risk of peroxidation, and last but not least, demanding synthesis using its gaseous and also toxic and highly flammable monomer ethylene oxide. In this study, we employed the *grafting from* approach to construct hydrophilic polyglycidol chains using the multiple resorcinolic hydroxyl groups of the CRA and Noria oligomers. Polyglycidol (PG) has been considered as an alternative to PEG by which the disadvantages of the latter have been sought to circumvent. PG can be prepared with different chain topology—branched and linear. The former is obtained by anionic polymerization of glycidol, whereas the synthesis of the latter starts from anionic polymerization of glycidol derivatives (protected glycidols) and the subsequent cleavage of the protecting group. In general, PG (branched or linear) exhibits equal or even higher biocompatibility compared to PEG and displays enhanced hydrophilicity and functionality because of the numerous hydroxyl groups.

The present study introduces a strategy for the synthesis of water-soluble polymer-derivatized calix resorcinarene ladder oligomers with open chain and cyclic structures comprising branched or linear polyglycidol segments/arms by using the *grafting from* approach. Further, we report on the characterization and preliminary biological screening of the novel structures.

## 2. Materials and Methods

### 2.1. Materials

Resorcinol (ACS Reagent, ≥99.0%, Sigma-Aldrich, St. Louis, MO, USA), 2,5-dimethoxytetrahydrofuran (DTHF) cis- and trans (98% from Alfa Aesar, Haverhill, MA, USA), glycidol (2,3-epoxypropanol, 96%, Aldrich), ethyl vinyl ether (99%, Aldrich), p-toluenesulfonic acid (ACS reagent, ≥98.5%, Sigma-Aldrich), dimethyl sulfate (≥99%, Sigma-Aldrich), dimethylformamide (DMF) for UV analysis, methanol (anhydrous, 99.8%, Sigma-Aldrich), ethanol (96%, EMSURE^®^ Reag. Ph Eur, Sigma-Aldrich), 36% hydrochloric acid (Valerus Ltd., Sofia, Bulgaria), anhydrous K_2_CO_3_ (Valerus Ltd.), sodium sulfate (≥99.99%, trace metals basis, anhydrous, Sigma-Aldrich), AlCl_3_·6H_2_O (99%, Sigma-Aldrich), sodium hydrogen carbonate (anhydrous, ≥99.7%, Sigma-Aldrich), trifluoroacetic acid (Aldrich 99%), sodium hydroxide (reagent grade, pellets, ≥97%, Aldrich), chloroform (99%, Sigma-Aldrich) and glutaraldehyde 50 wt.% in H_2_O (Aldrich) were used as received. Diethyl ether (ACS Reagent ≥99.8%, Sigma-Aldrich) and tetrahydrofuran (>99.5% Fisher Scientific, Waltham, MA, USA) were distilled over sodium metal, and dichloromethane (ACS Reagent ≥99.9%, Sigma-Aldrich) and tert-butanol (anhydrous, ≥99.5%, Sigma-Aldrich) were distilled over CaH_2_. Potassium tert-butoxide 0.178 M solution in tert-butanol was prepared by dissolving 70 mg potassium metal in 5 mL anhydrous tert-butanol under argon and diluting the solution to 10 mL. Deionized water was obtained using a Millipore MilliQ system and additionally filtered through a 220 nm PTFE filter and a 20 nm cellulose filter.

### 2.2. Syntheses

#### 2.2.1. Synthesis of 3-Methoxyphenol and Ethoxyethyl Glycidyl Ether

3-Methoxyphenol and ethoxyethyl glycidyl ether (EEGE) were prepared following the synthetic protocols of Becker et al. [33] and Fitton et al. [34], respectively. The synthetic procedures are described in the Supplementary Materials.

#### 2.2.2. Synthesis of Macrocyclic Noria Oligomer

The synthesis of Noria oligomer was carried out according to the protocol of Niina et al. [35]. Briefly, 0.25 mL (0.236 g, 1.118 mmol, 1.4 eq) of glutaraldehyde and 1 g (7.937 mmol, 1 eq) of 3-methoxyphenol were dissolved in 1.5 mL CHCl_3_, and 1 mL of TFA was added to the resulting solution. The mixture was stirred at reflux for 48 h, during which the color turned red. The reaction mixture was cooled and poured into ice cold methanol. The resulting precipitate was collected and washed a few times with methanol and dried in vacuo, giving a white powder. Yield: 341 mg, 27.8%. ^1^H NMR (600 MHz, DMSO-d6): δ (ppm) 9.63–8.03 (phenolic -OH), 7.58–6.10 (aromatic H), 4.86–4.00 (>CH-), 3.97–3.45 (-OCH_3_), 3.03–0.46 (-CH_2_CH_2_CH_2_-).

#### 2.2.3. Synthesis of CRA Ladder Oligomer

Calix[4]resorcinarene (CRA) open structure ladder polymer was synthesized following the procedure described elsewhere [18] by a condensation–cyclization reaction of resorcinol and in situ generated 1,4-butanedial. Briefly, 4.4 g (20 mmol) of resorcinol were first dissolved in 12 g of 96% ethanol. Next, 1.6 g (6 mmol) of DMTHF and 4 g of 36% HCl were mixed and stirred at 80 °C for 24 h. The yellow-colored precipitation/soluble products mixture was then poured in a mixture of 200 mL of 96% ethanol and 10 mL of diethyl ether to fully precipitate the CRA oligomer by subsequent centrifugation at 4000 rpm for 15 min. Then, the precipitate was further re-dispersed in 50 mL of methanol by laboratory vortex and again centrifuged until an almost clear supernatant and pale-yellow precipitate formed. The final concentrated wet precipitate was again re-dispersed in 25 mL of absolute ethanol and vacuum filtered by 0.2 µm PVDF filter and dried under vacuum at 40 °C for 6 h resulting in a pale-yellow fine powder of purified CRA oligomer. Yield: 64%. ^1^H NMR (600 MHz, DMSO-d6): δ (ppm) 9.23–8.25 (phenolic -OH), 8.02–5.62 (aromatic H), 4.46–3.86 (>CH-), 2.42–0.86 (-CH_2_CH_2_CH_2_-).

#### 2.2.4. Synthesis of Noria-linPG

##### Noria-PEEGE

Thirty milligrams (0.016 mmol, 1 eq) of Noria was dissolved in 3 mL of anhydrous THF under argon and 1 mL of a 0.178 M solution of potassium tert-butoxide in tert-butanol (0.178 mmol, 11 eq) were added. The solution was cooled with liquid nitrogen and sublimated under vacuum. Then, 1.470 mL (1.306 g, 8.933 mmol, 550 eq) of EEGE were added through a syringe and the mixture was heated at 90° C for 72 h. Afterward, the reaction mixture was cooled to room temperature, polymerization was terminated by adding 5 mL of methanol, and the reaction product was suspended in 100 mL of water. The suspension was extracted several times with DCM, the extracts were dried with anhydrous MgSO_4_, filtered, and the solvent was evaporated at reduced pressure. The residue was dialyzed against 15 kDa cellulose membrane in a mixture of 10:1 methanol to water. Yield: 650 mg, 54.5%. ^1^H NMR (600 MHz, CDCl3): δ (ppm) 6.9–5.9 (aromatic H) 4.90–4.60 (-CH(CH_3_)-O-CH_2_-CH_3_), 4.02–3.14 (-O-CH_2_-CH(CH_2_-O-)-O- and -CH(CH_3_)-O-CH_2_-CH_3_), 1.40–0.99 (-CH(CH_3_)-O-CH_2_-CH_3_.

##### Noria-linPG

To 150 mg of Noria-PEEGE dissolved in 15 mL of methanol, 0.1 mL of TFA were added, and the solution was left overnight at room temperature and gentle stirring was performed. The mixture was then evaporated to dryness yielding a pure product. Yield: 78 mg, 100%. ^1^H NMR (600 MHz, DMSO-d6): δ (ppm) 7.28–6.10 (aromatic H) 4.90–4.14 (-OH), 3.74–3.03 (-O-CH_2_-CH(CH_2_-O-)-O-).

#### 2.2.5. Synthesis of CRA-brPG

The dispersal of 0.1 g (0.04 mmol) of CRA ladder oligomer was performed by short ultrasound treatment in 10 g of DMF until full dissolution of CRA. Then, 0.13 g (0.94 mmol) of anhydrous K_2_CO_3_ was added and the mixture was stirred under argon at 60 °C for about 30 min until full dissolution of alkalized CRA was achieved, resulting in a red-colored solution. The resultant solution was filtered through a 5 μm Teflon filter in order to remove the insoluble KHCO_3_ formed. Next, the alkalized CRA filtrate was poured into a 25 mL three neck round bottom flask equipped with a reflux condenser and heated at 80 °C under an argon atmosphere. Then, 1 g of glycidol was drop-wise added to the alkali CRA solution and stirred at 500 rpm for 1 h. After that, another portion of 1.4 g of glycidol was slowly added and the reaction mixture was left for another 20 h. The progress of polyglycidol grafting onto hydrophobic CRA was monitored by simply taking small aliquots from the reaction mixture at one-hour intervals and pouring them into an acidic 2M hydrochloric solution (pH = 1), where the unreacted and slightly functionalized CRA core quickly precipitated. After six hours of reaction time, the precipitate became visibly looser, resulting in a completely soluble product after 12 h of reaction time. Next, the DMF solvent from the reaction mixture was rotary vacuum evaporated at 70 °C and under 5 mbar of pressure, resulting in a honey-like viscous product. The isolated product was further dissolved in 10 mL of deionized water and acidified with one drop of conc. HCl solution to pH 2 turning its color into pale-yellow. The excess HCl was neutralized with the addition of small amount of NaHCO_3_ after which the aqueous concentrate was further purified from unreacted glycidol, remained DMF, neutralization salts and the fraction of oligomeric homopolyglycidol with the use of a semi-permeable cellulose acetate membrane with MWCO 2000 g/mol and distilled water as a dialysis medium. The purified CRA-brPG aqueous solution was vacuum concentrated until the full removal of water was achieved, resulting in a pale-yellow viscous product soluble in the whole pH range. ^1^H NMR (600 MHz, DMSO-d6): δ (ppm) 7.28–6.10 (aromatic H) 4.90–4.14 (-OH), 3.74–3.03 (-O-CH_2_-CH(CH_2_-O-)-O-).

### 2.3. Methods

#### 2.3.1. Nuclear Magnetic Resonance (^1^H NMR)

^1^H NMR measurements were conducted on a Bruker Avance II spectrometer (Billerica, MA, USA) operating at 600 MHz and Bruker Avance III operating at 400 MHz using CDCl_3_ or DMSO-d6 at 25 °C.

#### 2.3.2. Gel Permeation Chromatography (GPC)

The molar mass characteristics of THF soluble Noria oligomer and Noria-PEEGE protected copolymer were determined by GPC. The GPC analysis was performed on a Shimadzu Nexera XR LC20ADXR liquid chromatograph with a DGU-20A5R degasser, a SIL-20ACHT autosampler, an UV/Vis SPD-20A detector, and a RID-20A refractive index detector. A set of three PLgel (Agilent Tech., Shropshire, UK) GPC columns: 10 μm PL gel mixed-B, 5 μm PL gel 500 Å, and 50 Å were used for the chromatographic separation of the products. Tetrahydrofuran (THF) was used as an eluent at a flow rate of 1 mL/min at 40 °C. Samples were prepared as solutions in THF. Molar mass characteristics of the copolymers were calculated using a calibration curve constructed with monodisperse polystyrene standards. Data acquisition and processing were performed using LabSolutions v.5.54 GPC software. For the purpose of clarity, the GPC eluograms are shown in the range in which meaningful signals are observed.

#### 2.3.3. Matrix-Assisted Laser Desorption/Ionization—Time of Flight (MALDI-TOF)

MALDI-TOF analyses were performed using a Shimadzu AximaConfidenseTMmass spectrometer (Kratos Analytical Ltd., Manchester, UK) equipped with an ionization source of variable ion extracting energy (linear +25 kV/−20 kV, reflectron +20 kV/−20 kV) working in positive and negative ion operation, linear flight tube of 1.2 m drift length and reflectron effective 2.0 m drift length and detectors: linear mode—multiple dynodes and reflectron mode—fast micro-channel plate. All experiments shown here were made in the linear-positive-ion mode due to the increased sensitivity in this range analyzing relatively low molar masses of the investigated (co)polymers. Samples were prepared by dissolving CRA and Noria oligomers in DMF and THF, respectively, and α-cyano-4-hydroxycinnamic acid (αCHCA) in diluent for matrix (MALDI-TOFMixTM kit, LaserBio Labs, Valbonne, France) or 2,5-dihydroxybenzoic acid (DHB)) in THF and a cationizing agent (Li salt dissolved in acetonytrile/water) at a polymer/matrix/salt ratio of 10:10:1. Noria-linPG and CRA-brPG were dissolved in THF or DMF, respectively, and mixed with DHB matrix and cationizing agent in the same ratio. Ions were formed by laser desorption at 337 nm N_2_ laser (3 ns pulse width, 100 mj per laser shots, 50 Hz maximum pulse rate) and accelerated with +25 kV (positive ion operation). All mass spectra were collected, baseline corrected and smoothed by the Shimadzu Biotech Launchpad 2.9.9.3 software. Molar mass calculations were made by the data system using the Kratos Analytical Polymer v.2.9 software. For the purpose of clarity, the mass spectra are shown in the range in which meaningful signals are observed.

#### 2.3.4. Analytical Ultracentrifugation (AUC)

CRA-brPG and Noria-linPG were subjected to analytical ultracentrifugation (AUC) analysis. For these experiments, polymer water solutions at a concentration range of 0.2 to 1 mg/mL were used. Sedimentation velocity (SV) experiments were performed at 20 °C on a ProteomeLab XL-I (Beckman Coulter, Brea, CA, USA) analytical ultracentrifuge, equipped with a UV–Vis absorption optical system. The absorbance of the samples was monitored at 280 nm. A rotor speed of 40,000 rpm was selected for all measurements. A single scan at 3000 rpm was run before the high-speed SV investigations as a radial calibration to check the cell assembly, obtain the absorbance curve depending on the wavelength at a fixed radius, and eliminate any possible higher molar mass impurities. Subsequently, 150 radial scans in time intervals of 4 min were recorded, thus providing the absorbance variation as a function of radial displacement in the solution. The sedimentation coefficient distributions were determined by the continuous c(S) distribution model solving Lamm equation on the SEDFIT software (v. 16.1). The SEDFIT software uses the obtained sedimentation coefficient to determine the corresponding effective reduced molar mass using the Svedberg equation (Equation (1)):

S = MD(1 − υρ)/RT(1)

where M, D, R, T, υ, and ρ are the molar mass, diffusion coefficient, gas constant, absolute temperature, partial specific volume, and density of the solvent, respectively. Sedimentation equilibrium runs were analyzed with nonlinear exponential fit for single species to give a weight average molar mass.

#### 2.3.5. Static Light Scattering (SLS)

SLS measurements were carried out in the interval of angles from 40 to 140° at 25 °C using Brookhaven BI-200 goniometer with vertically polarized incident light at a wavelength λ = 633 nm supplied by a He−Ne laser operating at 35 mW. The SLS data were analyzed using the Zimm, Debye, and Berry plot formalisms. The latter yielded values of the static light scattering parameters of the lowest uncertainty; therefore, SLS data are presented by the Berry plot. In the Berry plot method, information on the weight-average molar mass, M_w_, was obtained from the dependence of the quantity (Kc/R_θ_)^1/2^ on the concentration (c) and scattering angle (θ). Here, K is the optical constant given by K = 4π^2^n_0_^2^(dn/dc)^2^/N_A_λ^4^, where n_0_ is the refractive index of the solvent, N_A_ is Avogadro’s constant, λ is the laser wavelength, and R_θ_ is the Rayleigh ratio at angle θ. dn/dc is the refractive index increment measured in water in separate experiments on an Orange GPC19 DNDC refractometer. The dn/dc values of the investigated systems were in the 0.240−0.242 mL/g range.

### 2.4. Biological Evaluation

#### 2.4.1. Cell Cultures and Cytotoxicity Assessment

Three cell lines were used to assess both cytotoxicity and the possibility of a cell-specific response. Two of the cell lines were normal, non-tumorous—a diploid human skin fibroblast cell line HFF (at 18–22 passages) and the spontaneously immortalized human keratinocyte cell line HaCaT, which has the ability to differentiate in culture and is widely used as a model of skin epithelium in vitro. The results of these lines were compared with HepG2 hepatocarcinoma, which is widely used to evaluate cytotoxicity and hepatotoxicity of xenobiotics. Cells were grown in standard conditions according ATTC, in DMEM or DMEM F-12 Ham with 10% (*v*/*v*) FCS, streptomycin (0.1 mg/mL), penicillin (0.06 mg/mL) at 37 °C in a 5% CO_2_ water-saturated atmosphere. For MTT experiments, cells were plated at an initial concentration of 5 × 10^4^ for 96 well plates.

A cytotoxicity assessment of polymer particles was conducted by MTT assay, as previously described [36]. The absorbance was measured at 570 nm using an Epoch Microplate Spectrophotometer, BioTek^®^ Instruments Inc., Winooski, VT, USA with the Gen5™ Data Analysis software, version 1.11.5. Data were presented as a percentage of the mean control value (the absorbance of untreated cells).

#### 2.4.2. Flow-Cytometry Analysis of Cell Proliferation and Apoptosis

Cell cycle and apoptosis analyses were performed using a Guava^®^ easyCyte™ Flow Cytometry system (Luminex, Austin, TX, USA). For the assessment of changes in cell cycle Guava^®^ Cell Cycle Reagent, Luminex was used according to the manufacturer’s instructions. Briefly, cells were treated with aqueous solutions of the investigated samples with increasing concentration from 5 to 300 µg/mL for 72 h. Untreated cells (negative) and cells treated with 0.1 µg/mL colcemid for 4 h (positive) were used as controls. After treatment, the cells were washed with sterile PBS, trypsinized, and fixed with ice-cold 70% ethanol in Eppendorf tubes. Cells were stained as described by the manufacturer and the cell cycle data of each sample were acquired on the Guava easy Cyte instrument. The distribution of cells in the cell cycle phases was performed depending on the DNA content in each cell.

For the assessment of induction of apoptosis, Guava Nexin^®^ Reagent (Luminex) was used according to the manufacturer’s instructions. Briefly, cells were treated for 72 h, as previously described, collected by trypsinization (cells from media before trypsinization were also collected and added to the respective samples) and stained for 30 min with Guava Nexin^®^ Reagent. For the correct reading of results, four types of controls were used—negative control from untreated cells, early apoptotic cells (treated for 30 min with 1 mM H_2_O_2_), late apoptotic cells (maintained 24 h in FCS-free media), and cell debris (treated with 0.1% Triton X-100, immediately before staining).

## 3. Results and Discussion

### 3.1. Synthesis and Characterization of Ladder CRA and Noria Oligomers

**Scheme 1:**
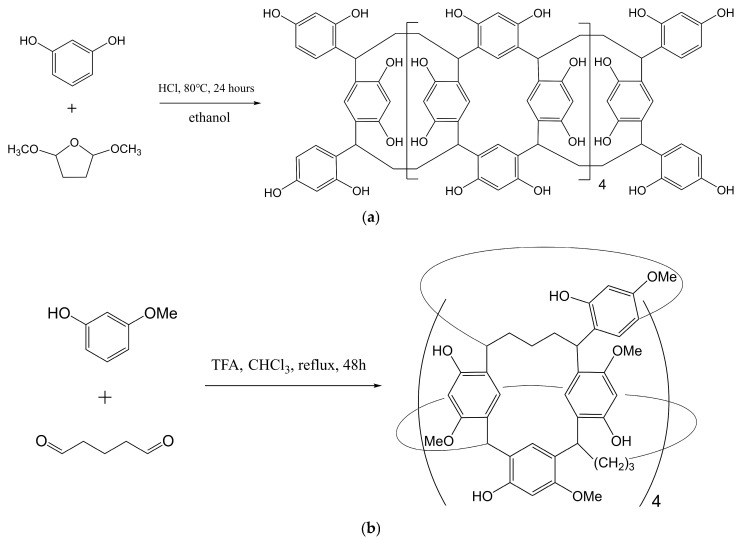
Synthesis of ladder CRA (**a**) and Noria (**b**) oligomers.

For the synthesis of the CRA ladder oligomer with open chain structure, we used a slightly modified original methodology published by Kudo et al. [19] where the condensation–cyclization reaction of resorcinol and in situ generated 1,4-butanedial from the decomposition of dimethoxy tetrahydrofuran in the presence of HCl takes place (Scheme 1a). After 24 h, the reaction mixture was precipitated twice, redispersed in absolute ethanol, vacuum filtered, and dried in vacuo.

**Figure 1:**
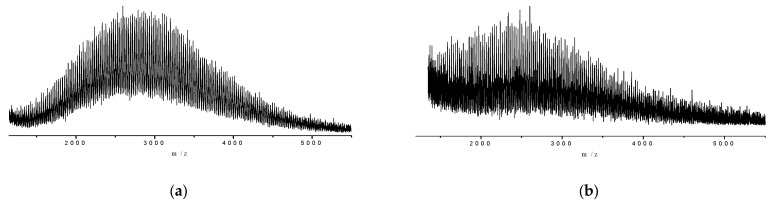
MALDI-TOF spectra of CRA (**a**) and Noria (**b**) oligomers.

**Figure 2:**
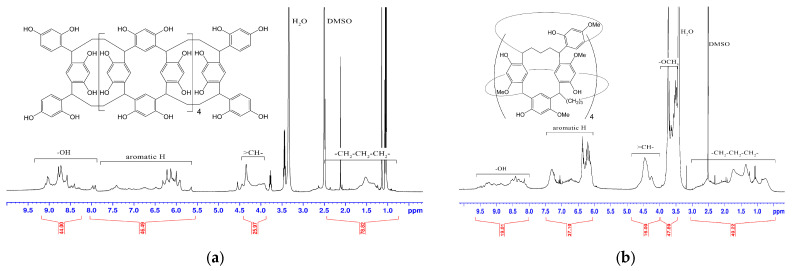
^1^H NMR spectra CRA ladder (**a**) and Noria (**b**) oligomers in DMSO-d6.

As the product is soluble only in aprotic highly polar solvents but not soluble in the commonly used solvents for GPC (e.g., THF), MALDI-TOF was used to determine the molar mass of the synthesized oligomer. The mass spectrum is shown in Figure 1a. The well-resolved spectrum indicated the formation of an oligomeric product with narrow molar mass distribution. The calculated number-average molar mass of 2300 g/mol corresponds to an average degree of polymerization (DP_n_) of 5.3. Molar masses and molar mass distribution (M_w_/M_n_) are summarized in Table 1. The obtained value of 432 g/mol for the molar mass of the monomer unit (M_unit_) matches the theoretical one of the CRA ladder oligomer product obtained by the condensation reaction of resorcinol and 1,4-butanedial. The composition of the CRA ladder oligomer was determined from the ^1^H NMR data. A representative spectrum is shown in Figure 2a. The ^1^H NMR spectrum showed signals for methylene (0.8–2.5 ppm), methine (4.1–4.4 ppm), aromatic (5.6–7.8 ppm), and hydroxyl (7.9–9.3 ppm) protons. A FTIR and UV–vis spectra of CRA are shown in Figures S1a and S3a and the main bands and absorbances are identified in the Supplementary Materials.

The condensation of 3-methoxyphenol, prepared by monomethylation of resorcinol with dimethyl sulfate [33], with glutaraldehyde was carried out according to a procedure described by Niina et al. [35] to obtain the cyclic product, Noria (Scheme 1b). The resulting Noria cyclic oligomer in which half of the hydroxyl groups are methoxylated was characterized by ^1^H NMR, FTIR, and a UV spectroscopy. Representative spectra are shown in Figure 2b and in the Supplementary Materials. New resonances at 3.5–4.0 ppm, assigned to the methyl protons of -OCH_3_ groups, along with those of the methine, methylene, aromatic and hydroxyl protons, appeared in the ^1^H NMR spectrum (Figure 2b). For the cyclic oligomer Noria, the main absorption bands are 3335 cm^−1^ phenolic O-H stretching, 2931 cm^−1^ C-H stretching, 1606 cm^−1^ C=C stretching, 1196 cm^−1^ and 1138 cm^−1^ C-O stretching vibrations as shown in Figure S2a. The UV–vis spectrum of the product is shown in Figure S3b. Molar mass characteristics were obtained from MALDI-TOF. The mass spectrum and calculated molar mass data are shown in Figure 1b and Table 1, respectively. As in the case of the CRA ladder, a well-defined symmetrical spectrum is observed. The value of DP_n_ obtained from the MALDI-TOF analysis showed a value close to 4 (DP_n_ = 4.3), suggesting formation of a predominantly four-member cycle. The calculated M_unit_ = 488 g/mol of synthesized via condensation of 3-methoxyphenol with glutaraldehyde Noria oligomer corresponds well to theoretically predicted molar mass of the monomer unit.

**Figure 3:**
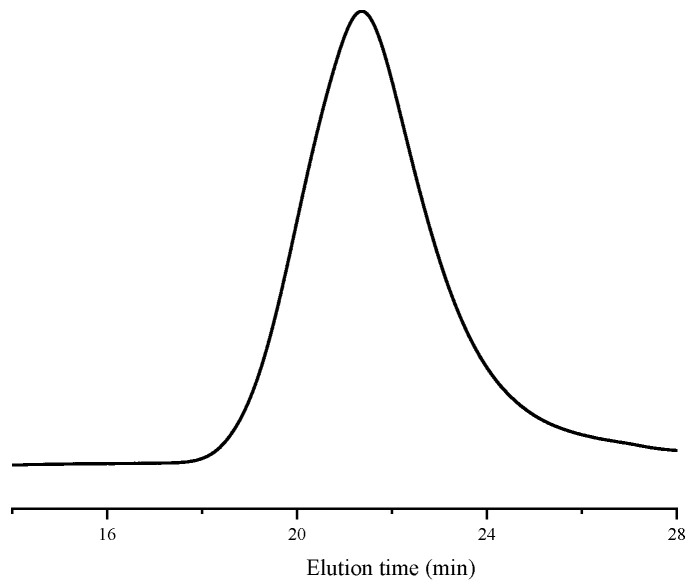
GPC trace of Noria taken in THF.

Expectedly, the Noria product exhibited solubility in common organic solvents, such as THF, apparently owing to the significant reduction in the intermolecular hydrogen-bonding effect between hydroxyl groups upon methoxylation. This improved solubility allowed us to use the GPC method for determination of the molar mass characteristics of the Noria oligomer. The GPC curve (Figure 3) showed a monomodal slightly broad distribution, similar in shape to that obtained by mass spectrometry (cf. Figure 1b and Figure 3), which was a confirmation of the accuracy of the mass spectral data. The observed drift (tailing, sweeping) of chromatographic peak resulting in broadening of dispersity index can be attributed to the absence of suboptimal chromatographic conditions, namely partial insolubility in the eluent and/or interaction with the packing of columns. The derived molar mass values were slightly lower than those from MALDI-TOF (Table 1), but it must be noted that these were relative to polystyrene standards.

In summary, by following well established procedures [18,19,35] with slight modifications, two oligomeric *ladder* type products of different structures and compositions were obtained: linear with DP_n_ of ca. 5 (CRA) and cyclic with DP_n_ of ca. 4 (Noria). Considering their chemical structures (Scheme 1) and degrees of polymerization, the average numbers of hydroxyl groups per molecule of CRA and Noria of 44 and 16, respectively, were determined.

### 3.2. Synthesis and Characterization of Polyglycidol-Derivatized CRA and Noria Oligomers

**Scheme 2:**
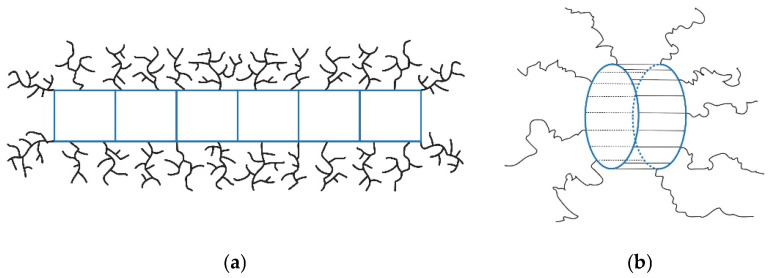
Schematic presentation of the molecular topology of polyglycidol-derivatized CRA (**a**) and Noria (**b**) oligomers.

To synthesize polyglycidol-derivatized CRA and Noria oligomers, we selected the *grafting from* approach by which monomers are covalently bonded and polymerized as side chains onto the backbone. The two ladder oligomers have numerous hydroxyl groups that can be easily converted into initiating centers for polymerization of oxirane monomers. The oligomers, however, strongly differ in molecular structure: whereas the molecule of the oligomer with open chain structure (CRA) can be considered as two-dimensional, flat and rigid with two distinguished hydrophobic surfaces, that of the cyclic analog (Noria) is clearly three-dimensional with a “*hidden*” inner surface in the interior forming a hydrophobic fixed hole in the center. Furthermore, CRA is characterized with more numerous hydroxyl groups of higher density than those of Noria. Therefore, to achieve high grafting density and full coverage of the CRA molecule, it is reasonable to use a small, not bulky monomer, whereby (i) the steric hindrance, posed by the high density of hydroxyl groups, could be reduced and (ii) branching of the propagating chains upon polymerization could be triggered. On the other hand, due to the less numerous and of lower density hydroxyl groups, the steric hindrance in Noria is lower, which allows the use of bulkier monomers such as EEGE or other protected glycidols to construct linear polyglycidol chains. We hypothesize that by using these approaches the topology of the initial CRA molecule (2D, flat) would be preserved, whereas the “water wheel”-like structure of the cyclic oligomer would evolve into a star-like chain architecture. The hypothesized molecular topologies of the polyglycidol-derivatized ladder oligomers are presented in Scheme 2. The target total molar masses of the PG moieties ranged 40–45 kg/mol, being slightly higher for the planar oligomer (CRA) because of its more hydrophobic character.

#### 3.2.1. Synthesis and Characterization of CRA-brPG

**Scheme 3:**
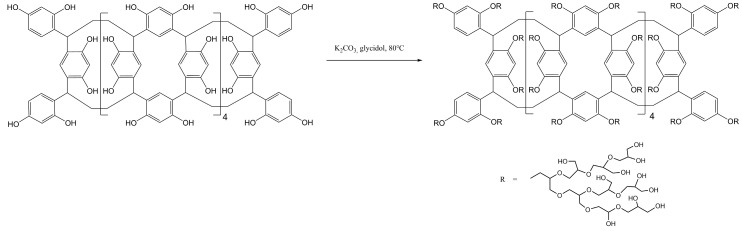
Synthesis of CRA-brPG.

CRA ladder oligomer, containing an average of 44 hydroxyl (resorcinic OH) groups, was partially (approx. 50%) deprotonated by a reaction with anhydrous K_2_CO_3_ in DMF and used as an initiator for the polymerization of glycidol. In the anionic polymerization of glycidol, branching is inevitable because the primary and secondary side groups in the polymer chain can be in situ converted into propagating alkoxide species [37,38]. Noteworthy, we employed a slow monomer addition strategy [37,39,40] to obtain a product with a narrow molar mass distribution and to minimize the formation of homopolyglycidol, that is, branched polyglycidol chains/macromolecules that are not grafted on the CRA ladder scaffold. The reactions (formation of the initiator and polymerization of glycidol) are shown in (Scheme 3).

**Figure 4:**
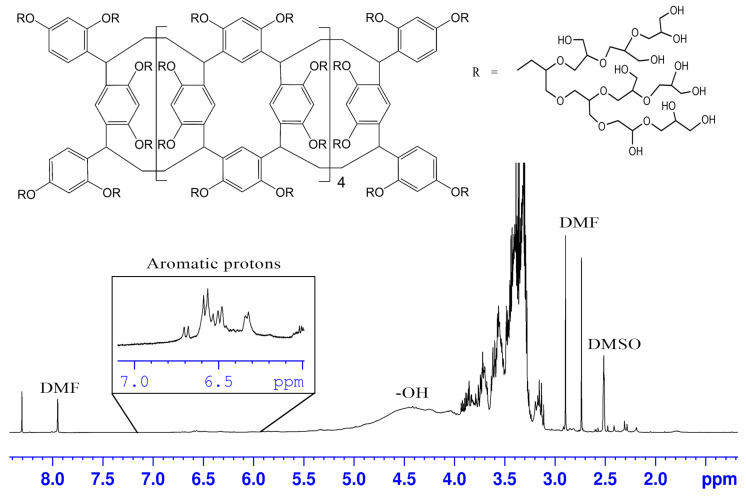
^1^H NMR spectrum of CRA-brPG in D_2_O.

In order to isolate the product (hereinafter CRA-brPG), the reaction mixture was first continuously vacuum dried in order to remove as much of the solvent as possible and, in the next steps, the concentrated honey-like mass was dissolved in water, acidified to pH 2 with 1M HCl to neutralize any remaining resorcinate or alkoxide end groups, and subsequently purified from KCl neutralization salt, unreacted glycidol, and homopolyglycidol fractions by dialysis membrane with MWCO of 2000 g/mol. The isolated aqueous dialysate solution of CRA-brPG was further concentrated and vacuum dried yielding pale-yellow viscous product. ^1^H NMR, FTIR, and UV–vis spectra of the product are shown in Figure 4, Figure S1b, and Figure S3a, respectively. The as-obtained product was found practically insoluble in anhydrous THF, which prevented us from running GPC for determination of its relative molar mass characteristics. Interestingly, we found the CRA-brPG was soluble in THF, containing about 2 wt.% of water. However, as the use of such a solvent in GPC analysis was contradictive, further molar mass characterization of this derivative was performed by MALDI-TOF, AUC, and SLS.

The theoretical total DP of the PG moieties, calculated from the material balance, was 608, corresponding to a molar mass of ca. 45,000 g/mol. Apparently similar values of the DP and molar mass were determined from the ^1^H NMR spectroscopy (Figure 4). The molar mass characteristics data of the CRA-brPG, determined with MALDI-TOF, are presented in Table 2. In addition, the molecular ions are resolved, and the composition of the product can be proposed. The MALDI-TOF spectrum showed multiple distributions that overlap (Figure S4). The Polymer Analysis feature was used to analyze the spectrum. When analyzing a polymer series, the program first attempts to find which peaks are part of the polymer series. The selected monomer mass was manually identified by selecting two adjacent peaks that are separated by one monomer mass. The difference between the masses of these peaks is used as a first approximation to the monomer mass. A peak is only considered a part of the same polymer series if it is separated in mass from another peak in the series by this monomer mass ± tolerance. The software repeats this analysis for every distribution that it finds within the spectrum, and finally Polymer Statistics displays the statistical information about the spectrum. Following this procedure, the molar masses of the two moieties of the CRA-brPG copolymer were calculated (Table 2). Comparing the molar mass data of the attached PG arms to the CRA ladder core in copolymer obtained from ^1^H NMR (and material balance) and MALDI-TOF analyses showed some difference, namely, lower values obtained with the MALDI-TOF methodology. One possible reason can be found in the production of highly branched PG chains, thus limiting the solubility of the high molar mass fractions. The latter, in turn, decreases the ability of the copolymer sample to co-crystallize with the matrix used in the MALDI-TOF assay.

**Figure 5:**
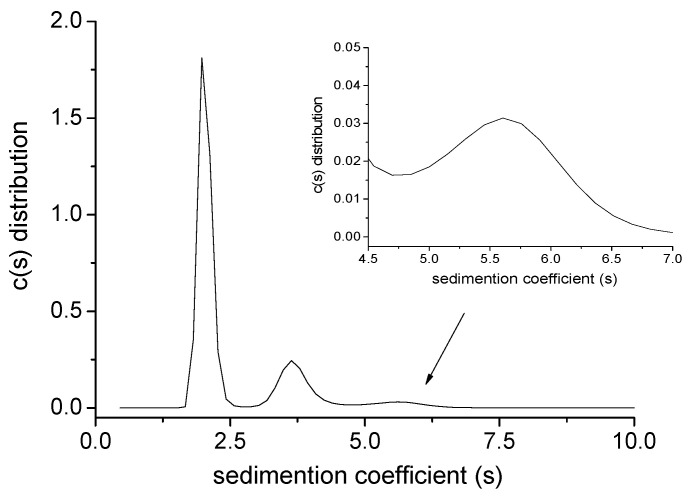
Sedimentation coefficient distribution from AUC measurements for CRA-brPG. The inset shows a scale-up in the region for s > 4.5 S. The corresponding molar mass values for each peak are shown in Table 2.

Through AUC, we gained further insights into the characteristics of CRA-brPG. Sedimentation velocity experiments were carried out for a 0.8 mg/mL aqueous solution at room temperature. The data were analyzed using the SEDFIT software, yielding sedimentation coefficient distribution. The sedimentation coefficient distribution is shown in Figure 5, whereas the corresponding molar mass values, calculated by the Svedberg equation, are summarized in Table 2. As seen, the sample exhibited three peaks indicating the existence of different populations in the solution. The first peak at about s = 1.97 S was equivalent to a molar mass of 18.5 kg/mol, which well corresponded to the value determined by MALDI-TOF (Table 2), whereas the molar mass of 43.7 kg/mol, equivalent to the peak at s = 3.64 S, to those obtained from the ^1^H NMR and the theoretical molar mass from the material balance. As far as the peak at s > 5.0 S (the inset in Figure 5) and the equivalent molar mass of 77.6 kg/mol (Table 2) are concerned, these most likely correspond to low (dimers, trimers) aggregates.

**Figure 6:**
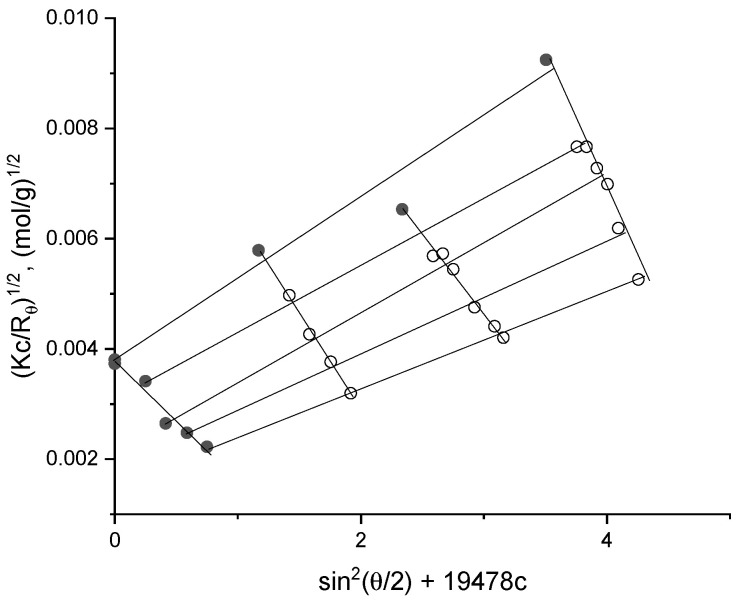
Berry plot of CRA-brPG in dilute aqueous solution at 25 °C: experimental point (open symbols); extrapolated points (closed symbols).

The formation of aggregates was confirmed by light scattering. SLS was performed in the concentration range 0.06–0.18 mg/mL. A representative Berry plot, from which the static light scattering parameters were determined, is shown in Figure 6. The derived weight-average molar mass, M_w_, of 69.0 kg/mol (Table 2) was consistent with the molar mass of the aggregates equivalent to the peak at s > 5.0 S in the AUC measurements (see above). Apparently, even in this very diluted concentration range, the scattering from the aggregates dominates. The attempts to observe monomolecular behavior and to characterize the individual (non-associated) macromolecules by light scattering (dynamic and static) by further diluting the solutions failed: the overall scattered light intensity was very weak, almost comparable to that of the solvent, yielding a very low signal-to-noise ratio with a large contribution of “dust”, which ruined the experiments.

In summary, the employed approach to graft branched polyglycidol chains from the CRA ladder scaffold yielded two fractions of different degrees of polymerization of the branched PG arms. This could be associated with the large number (44) and high density of hydroxyl groups of the CRA oligomer, leading to steric hindrance of the propagation. When the saturation (steric hindrance) is reached, the polymerization proceeds on the accessible active centers and the second (of higher DP of branched polyglycidol) fraction starts to form. Interestingly, the two fractions were detected separately by different methods, which yielded seemingly inconsistent results about the DP and molar mass of the product CRA-brPG. AUC, however, revealed their co-existence and allowed simultaneous characterization. In a very dilute regime in an aqueous solution, a fraction of aggregates was formed, as revealed by AUC and SLS. The aggregates were of seemingly low aggregation numbers—dimers, trimers. The origin and formation of aggregates is unclear. We can speculate on hydrophobic interactions considering the amphiphilic character of CRA-brPG or formation of layered structures held together by hydrogen bonds via the numerous OH groups of the branched PG chains.

#### 3.2.2. Synthesis and Characterization of Noria-linPG

**Scheme 4:**
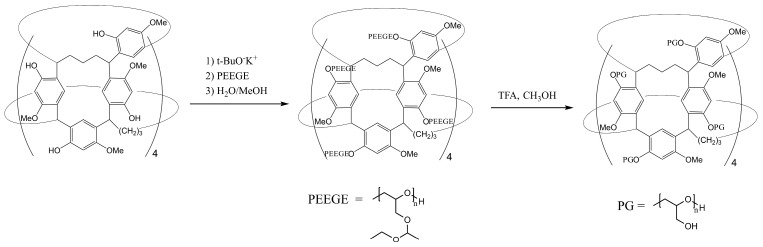
Synthesis of Noria-linPG.

To obtain linear polyglycidol, the hydroxyl group of the monomer should be protected thus rendering the former inert in the condition of anionic polymerization. The hydroxyl group was protected by reacting glycidol with ethyl vinyl ether in the presence of catalytic amounts of p-toluenesulfonic acid yielding ethoxyethyl glycidyl ether (EEGE) [34]. The synthesis of EEGE is shown in the Supplementary Materials. The anionic polymerization of EEGE has been shown possible, close to living [41], and applicable to obtain various (co)polymers [42,43,44,45,46,47]. To obtain Noria with linear PG chains (Noria-linPG), a two-step procedure involving (i) anionic polymerization of EEGE and (ii) cleavage of the protective groups was applied (Scheme 4). Noria was converted into an anionic macroinitiator by deprotonation of the phenol groups with potassium tert-butoxide. The reaction was performed in slight excess of Noria (11 eq alkoxide for 12 eq -OH groups) to ensure complete consumption of the tert-butoxide since it could compete in the act of initiation of the polymer chains.

**Figure 7:**
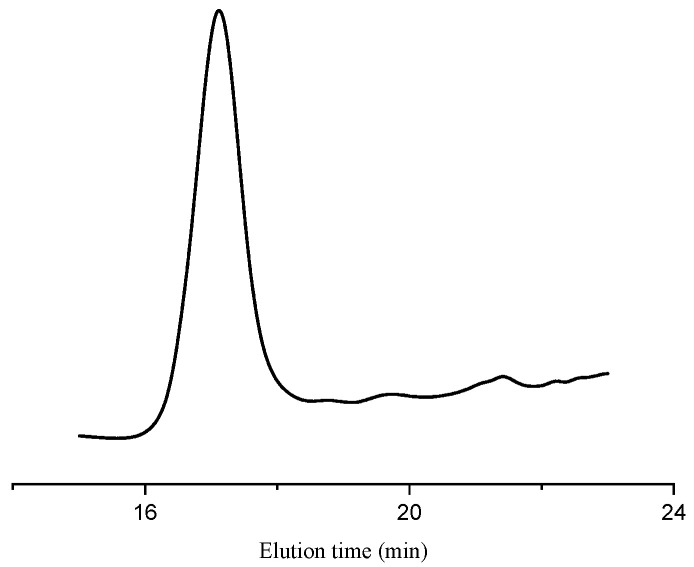
GPC traces tracked with an UV detector of the precursor Noria-linPEEGE taken in THF.

**Figure 8:**
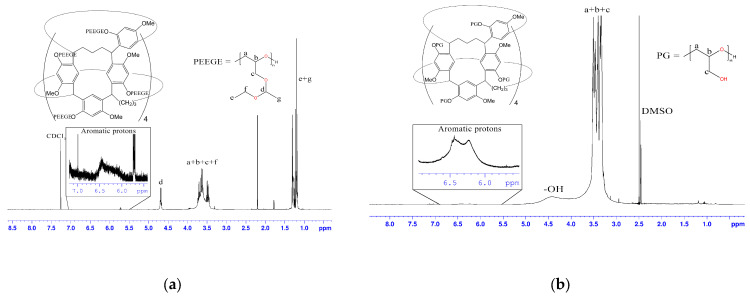
^1^H NMR spectra of Noria-PEEGE precursor in CDCl_3_ (**a**) and resulting Noria-linPG in DMSO-d6 (**b**).

The formation of PEEGE arms grafted from Noria was proved by FTIR, UV–vis (FTIR and UV–vis spectra of Noria-PEEGE is shown in Figures S2b and S3b in the Supplementary Materials), and GPC. The GPC traces of the starting Noria and the resulting Noria-PEEGE precursor are presented in Figure 3 and Figure 7. The GPC profiles showed monomodal major chromatographic peak spotted with a UV detector at a wavelength characteristic for Noria (see also Figure S3b), which is evidence for the successful grafting of PEEGE chains to the Noria core. The observed faint peaks in the eluogram at higher retention times, respectively, of lower molar mass can be attributed to the existence of small amounts of Noria-PEEGE of lower DP of PEEGE due to the interruption of the polymerization process as a result of partial inactivation of the growing species. The values of M_n_ and M_w_, relative to polystyrene standards, as well as molar mass distribution M_w_/M_n_ are summarized in Table 2. The real composition of the precursor was determined from the ^1^H NMR data. A representative spectrum is shown in Figure 8a. From the integral ratio of the aromatic H region (5.9 to 6.8 ppm) to acetal CH protons of the EEGE units (4.6 to 4.8 ppm) a total DP of PEEGE of 545 was determined, corresponding to the total molar mass of the PEEGE moieties of 79,600 g/mol (Table 2), which is in very good correlation with the theoretical molar mass and that from the material balance. In that aspect, the molar mass values from GPC may look inconsistently lower, but the results can be rationalized by recalling that (i) they were derived against linear polystyrene standards, that is, they are *relative* rather than *absolute* values and (ii) the molecular structure of Noria-PEEGE (Scheme 4), which, as other nonlinear polymers (star-like, hyperbranched, dendritic), is characterized with a considerably lower hydrodynamic size and, hence, resulting molar mass than their linear analogs [48,49,50]. Nevertheless, the molar mass distribution (M_w_/M_n_ = 1.28, Table 2) is acceptable, implying good control over grafting of the PEEGE chains from the Noria core.

A MALDI-TOF spectrum of Noria-PEEGE is presented in Figure S5, while calculated molar masses and molar mass distribution are shown in Table 2. As in the case of CRA-brPG, the observed total molar mass of the PEEGE moieties was lower than those taken from GPC and 1H NMR experiments. The lower MALDI-TOF values may be due to a poor dynamic range or detector fall off with mass [51]. In addition, the high molar mass end in the mass spectrum will disappear much earlier into the baseline noise than the high molar mass side of the GPC distribution, which means a few high molar mass molecules represent only a few ions in the mass spectrum but still show a significant bulk property as detected by the detector in the GPC apparatus.

**Figure 9:**
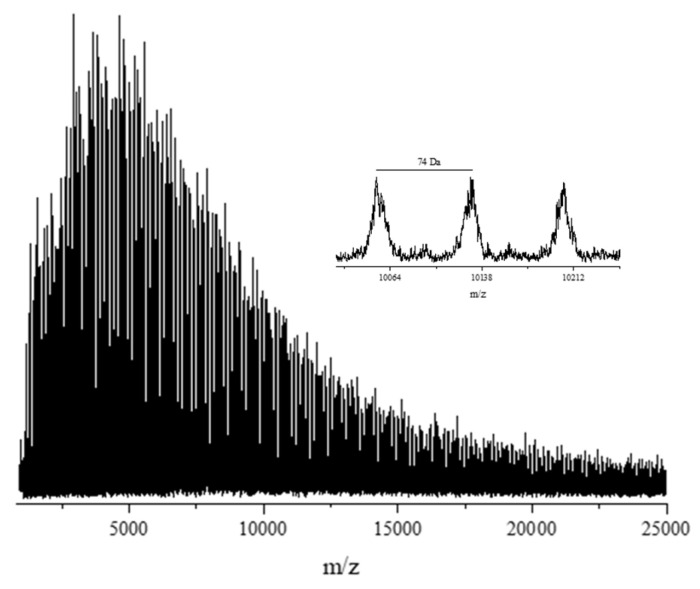
MALDI-TOF spectrum of Noria-linPG. The inset shows the difference between the peaks of 74 g/mol, which corresponds to the molar mass of the glycidol unit building the PG arms.

In the second step, the PEEGE chains were converted into PG chains by deprotection of the acetal groups, achieved by reacting the precursor with methanol in the presence of catalytic amounts of TFA (Scheme 4). The cleavage was successful, as evidenced by the difference in the FTIR spectra (Figure S2b,c) and the disappearance of the signals in the 1.0–1.5 ppm region in the spectrum of the resulting Noria-linPG (Figure 8b). The total DP of the PG moieties, determined form the ratio of aromatic protons at 6.0–7.1 ppm and methine and methylene protons of polyglycidol at 3.3–4.0 ppm, corresponded to a molar mass of 40.3 kg/mol (Table 2). The resulting product, Noria-linPG, was soluble in water in wide concentration ranges. It was further characterized by MALDI-TOF, AUC, and SLS. A MALDI TOF spectrum of Noria-linPG is presented in Figure 9, whereas the molar mass characteristics of the product are summarized in Table 2. It is immediately seen that the molar mass of Noria-linPG did not decrease proportionally to the reduction in the molar mass of the EEGE and glycidol monomer units (146 vs. 74, respectively) resulting from the release of the protective ethoxyethyl groups when compared to that of Noria-PEEGE. Different origins for mass discrimination in MALDI-TOF have been discussed in the literature and are associated with sample preparation that might ruin the homogeneous co-crystallization of polymer, matrix, and salt, on the one hand [52], and instrumental factors, on the other [53,54,55,56,57,58], i.e., the higher laser power can induce dimerization by clustering in the gas phase, yielding a molar mass distribution shift toward higher masses [56] or initiate unintended scissions in the polymer structure, as shown, for example, in the characterization of hyperbranched polyesters [59] and in the degradation of poly(methyl methacrylate) [60,61].

**Figure 10:**
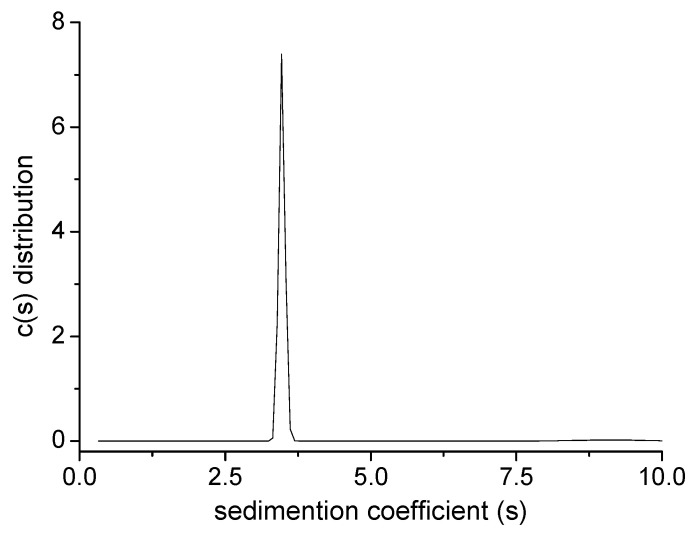
Sedimentation coefficient distribution from AUC measurements for Noria-linPG. The corresponding molar mass is shown in Table 2.

The AUC experiments for determination of the molar mass of Noria-linPG were carried out at the same conditions as for CRA-brPG—aqueous solution, room temperature, and slightly higher concentration of 1 mg/mL. The sedimentation coefficient distribution is presented in Figure 10. As seen Noria-linPG exhibited monomodal distribution represented by a single peak at s = 3.47 S, corresponding to a molar mass of 39.5 kg/mol (Table 2). The resulting value of the molar mass is in good agreement to that from the ^1^H NMR spectroscopy.

**Figure 11:**
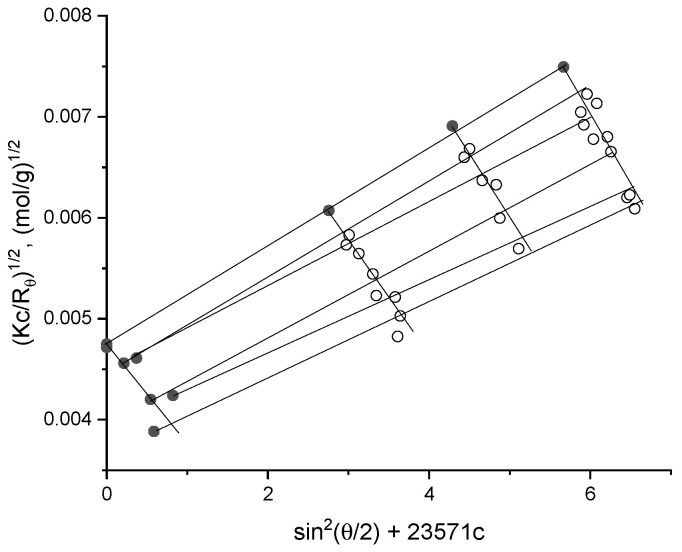
Berry plot of Noria-linPG in dilute aqueous solution at 25 °C: experimental point (open symbols); extrapolated points (closed symbols).

The weight-average molar mass, M_w_, of the product Noria-linPG was also determined by SLS. SLS was carried out in dilute aqueous solution in order to avoid any possible aggregation of the macromolecules and formation of large aggregates. SLS data were treated by the Berry plot method. A representative Berry plot is shown in Figure 11. As expected, an angular dependence of the quantity (Kc/R_θ_)^1/2^ was not observed because of the small size of the Noria-linPG macromolecules. The value of M_w_, derived from SLS, was 42,000 g/mol (Table 2), which is in good correlation with the results from ^1^H NMR and AUC. Very close (practically the same) values of M_w_ were obtained using Zimm plot and Debye plot methods for treating the SLS data. The value of the second virial coefficient, A_2_, was high (5.1 × 10^−2^ mL.mol/g^2^), indicating favorable particle/solvent interactions.

In summary, based on the results, obtained by different methods, we can conclude that the approach of grafting PEEGE arms from Noria cyclic ladder oligomer and their subsequent conversion into linear polyglycidol chains to obtain a water-soluble product is well controlled and yielded a well-defined product. The latter can be described as a star-like copolymer with 16 arms with an averaged DP of 34. The structure of Noria-linPG is schematically depicted in Scheme 2.

### 3.3. Biological Evaluation

#### 3.3.1. Biocompatibility

**Figure 12:**
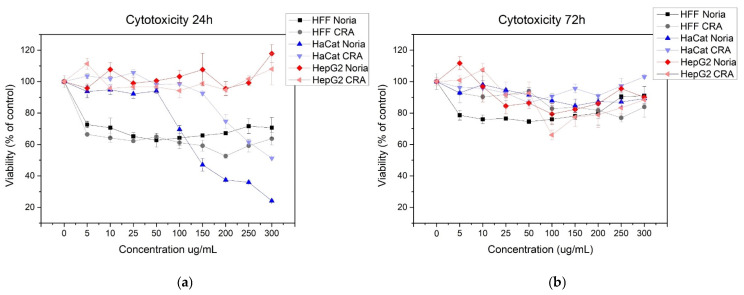
Viability of human cells 24 (**a**) and 72 (**b**) hours after treatment with polyglycidol-derivatized ladder oligomers CRA-brPG and Noria-linPG at a concentration of 5–300 µg/mL. The survival of control cells was taken as 100%.

The study of the biocompatibility of the two polyglycidol-derivatized ladder oligomers with different molecular topologies (see Scheme 2) was carried out on three cell lines of human origin—skin dipoid fibroblasts HFF, spontaneously immortalized keratinocytes HaCaT, and the hepatocarcinoma HepG2 cells, which retain many of the characteristics of active hepatocytes (Figure 12). In all three lines tested, CRA-brPG and Noria-linPG showed relatively low direct toxicity, with over 60% of cells surviving 24 h treatment with concentrations up to 300 µg/mL. Fibroblast cells were most sensitive, but with no pronounced concentration or particle type-specific response (Figure 12a). In keratinocytes, cytotoxicity was shown only at concentrations above 50 µg/mL, where there was a clear concentration dependence and Noria-linPG had a stronger effect on cell survival than CRA-brPG (Figure 12a). The observed decrease in viability in both cell lines was completely compensated after incubation for up to 72 h, which is a sign of adaptation of the cells (Figure 12b). We observed a different response of hepatocytes—at the beginning of treatment we had a signal exceeding that of controls, possibly due to increased mitochondrial activity as a result of stress, while at 72 h cell survival was slightly reduced and no adaptation was observed. The MTT test is a colorimetric assay based on the activity of NAD(P)H-dependent cellular oxidoreductase enzymes, which generate colored insoluble formazan by reducing 3-(4,5-dimethylthiazol-2-yl)-2,5-diphenyltetrazolium bromide. This process can be altered by changes in metabolic activity due to some cellular reactions to unusual events, such as polymer particles penetrating through the plasmalemma. Compensation of this increase in a subsequent period is an indication of adaptive changes.

#### 3.3.2. Cell Cycle and Apoptosis

To follow changes in the cell life cycle, we used two concentrations at which a cell survival of around 70% was observed—98 µg/mL and 213 µg/mL—and performed flow cytometric analysis. These concentrations were chosen according to the cytotoxicity shown at 24 h after treatment, which reflects direct toxicity without the possibility of adaptation. The retention of cells in the different periods of the cell cycle is an indicator of the potential impact of the different polymers, since clearly distinguishable consecutive biochemical processes take place in the individual periods: in the pre-synthetic, DNA replication is prepared and the amount of cytoplasm and cytoplasmic organelles increases; in the synthetic, DNA replication and centriole doubling are mainly carried out; and in the post-synthetic period, the construction of the dividing spindle and the exact separation of chromosomes between daughter cells are prepared. All of these processes are accompanied by a check and the possibility of arrest if disorders or damage are detected to ensure that only “healthy” undamaged cells are duplicated. The assessment of the distribution of cells in the main phases of the cell cycle was carried out by flow cytometric determination of the amount of DNA in an individual cell. In this case, cells that have not yet entered the synthetic period of interphase would have the normal amount of DNA for non-dividing cells, those that have completed replication would have double the amount, and cells that are in the synthetic period and are currently replicating DNA would be distributed between these two groups. The results for the distribution of cells in the main periods of the cell cycle are presented in Table 3.

All investigated variants resulted in human fibroblast cell cycle arrest in the post-synthetic G2 period, which may be associated with microtubule or cell membrane disturbances. Noria-linPG at a concentration of 213 µg/mL arrested the cell cycle of immortalized HaCaT and cancerous HepG2 cells in the pre-synthetic G1 period, which is a desirable effect in antitumor therapies. In all cell lines, Noria-linPG at the high concentration induced the most significant changes in the cell cycle. CRA-brPG at a concentration of 98 µg/mL caused cancer cells to be arrested in a post-synthetic G2 phase, and the higher concentration in a synthetic S phase, which may be a result of replicative stress.

**Figure 13:**
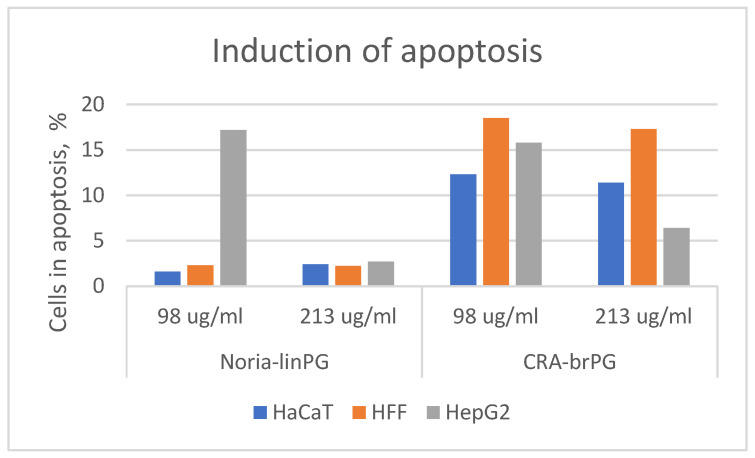
Induction of apoptosis after 72 h treatment with Noria-linPG and CRA-brPG.

Retention in the pre-synthetic and synthetic period could be a consequence of cellular stress, which, with longer exposure, could activate programmed cell death. It was also our motive to check for the induction of apoptosis in the longer treatment. The results are summarized in Figure 13. A comparison of the results of the treatment of the three cell lines with Noria-linPG at a concentration of 98 µg/mL is presented in Figure S6. Treatment with Noria-linPG at a concentration of 98 µg/mL resulted in the suppression of apoptosis compared to baseline levels reported in normal lines (keratinocytes and fibroblasts). Hepatocarcinoma cells showed significantly higher levels of apoptosis, indicating a selective action. The higher concentration of Noria-linPG resulted in a substantial suppression of apoptosis in all lines tested. CRA-brPG, on the other hand, induced higher percent of apoptosis in all tested lines. Keratinocytes and fibroblasts were more sensitive, while hepatocytes had a decreased induction of apoptosis as compared with treated with Noria-linPG at 98 µg/mL.

A large part of signaling pathways in cells originate from receptors on the cell membrane, which are included in the composition of the glycocalyx and are usually glycoproteins. Some of them, for example those that activate apoptosis, drive the relevant signaling pathway when they come together in groups (either by binding to ligands or by being incorporated into lipid rafts) and any intermolecular interaction leading to the stabilization of such groups would also lead to the activation of the corresponding process of cell suicide [62]. The different action of the studied polymer-derivatized ladder oligomers can be associated with their different molecular topology (Scheme 2). While the star-like Noria-linPG and its long linear chains of polyglycidol was found to disrupt membrane structures and interact directly with phospholipid bilayers, the flat CRA-brPG densely covered with short branched polyglycidol chains preferably interacted with the glycocalyx and cell surface receptors, leading to the activation of signaling pathways and apoptosis in keratinocytes and fibroblasts.

## 4. Conclusions

In this study, we report on the synthesis of water-soluble branched- and linear polyglycidol-functionalized ladder CRA and macrocyclic Noria-type oligomers. The core of calyx[4]resorcinarene ladder of open chain or macrocyclic Noria-type structure as well as their subsequent functionalization with linear or branched PG arms was accomplished in high yields starting from inexpensive and widely available reagents. The successful grafting of polyglycidol chains from the two types of ladder oligomers was confirmed by ^1^H NMR, FTIR and UV–vis spectroscopy and by GPC and MALDI-TOF measurements. AUC and SLS were used to assess the molar mass and degrees of polymerization of the polyglycidol moieties and overall composition. They revealed two fractions of different degrees of polymerization of the polyglycidol moieties and formation of a tiny fraction of low aggregates in an aqueous solution of CRA-brPG. A well-defined product of star-like molecular topology (Noria-linPG) was obtained by using the approach of grafting PEEGE arms and their subsequent conversion into linear polyglycidol. Preliminary biological evaluation revealed relatively low toxicity toward cell lines of human origin of CRA-brPG and Noria-linPG and their own biological activity: Noria-linPG exhibited an antitumor effect on hepatocarcinoma cells by arresting cell division in the pre-synthetic period and selectively activating apoptosis (at a concentration of 98 µg/mL); CRA-brPG induced apoptosis in all of the three cell lines tested, with diploid human fibroblasts being the most sensitive. These findings give hope for their successful future biomedical applications.

## Figures and Tables

**Table 1:** Molar mass characteristics of the CRA and Noria oligomers determined by GPC and MALDI-TOF.

Sample	GPC ^a^ (g/mol)		MALDI-TOF ^b^ (g/mol)			M_w_/M_n_
	M_n_ × 10^−3^	M_w_ × 10^−3^	M_n_ × 10^−3^	M_w_ × 10^−3^	M_unit_	
CRA	-	-	2.3	2.4	432	1.06 ^d^
Noria	1.7 ^e^	2.5 ^e^	2.1	2.8	488	1.47 ^c^, 1.32 ^d^

^a^ in THF; ^b^ in DMF; ^c^ GPC; ^d^ MALDI-TOF; ^e^ relative to polystyrene standards.

**Table 2:** Molar mass characteristics of the CRA-brPG, Noria-PEEGE, and Noria-linPG determined by ^1^H NMR, GPC, MALDI-TOF, AUC, and SLS.

Sample	^1^H NMR	GPC ^a^ (g/mol)		MALDI-TOF ^b^ (g/mol)			M_w_/M_n_	AUC (g/mol)	SLS (g/mol)
	M_n_ × 10^−3^	M_n_ × 10^−3^	M_w_ × 10^−3^	M_n_ × 10^−3^	M_w_ × 10^−3^	M_unit_		M_w_ × 10^−3^	M_w_ × 10^−3^
CRA-brPG ^b^	45.0	-	-	16.5 2.3	16.6 2.4	74 432	1.01 ^d^ 1.06 ^d^	18.5 43.7 77.6	69.0
Noria-PEEGE ^a^	79.6 ^c^	29.5	37.8	18.7 2.2	21.8 2.4	148 488	1.16 ^d^ 1.28 ^e^ 1.09 ^d^	-	-
Noria-linPG	40.3 ^c^	-	-	17.6 2.1	20.8 2.8	74 488	1.18 ^d^ 1.32 ^d^	39.5	45.0

^a^ in THF relative to polystyrene standards; ^b^ in DMF; ^c^ Only of PEEGE and PG moieties (i.e., excluding the molar mass of CRA and Noria; ^d^ MALDI-TOF; ^e^ GPC.

**Table 3:** Distribution of cells in cell cycle phases. Data are presented in percent of cells after 72 h treatment with Noria-linPG or CRA-brPG at concentrations of 98 µg/mL or 213 µg/mL. Cell clusters or polyploid cells are omitted.

Cell Line	Controls			Noria-linPG 98			Noria-linPG 213			CRA-brPG 98			CRA-brPG 213		
	G0/G1	S	G2/M	G0/G1	S	G2/M	G0/G1	S	G2/M	G0/G1	S	G2/M	G0/G1	S	G2/M
HaCaT	45.5	12.4	35.5	41.5	14.2	38.4	56.6	10.1	25.0	38.8	13.0	39.0	42.0	12.3	38.7
HFF	78.0	3.2	10.6	79.4	3.3	15.0	68.2	6.0	24.3	76.1	6.0	13.9	77.1	3.6	15.9
HepG2	59.8	10.8	19.8	55.9	12.7	22.8	65.0	8.6	10.2	51.5	9.5	25.3	57.2	16.6	12.6

## Data Availability

The original contributions presented in the study are included in the article, further inquiries can be directed to the corresponding author.

## Supplementary Materials

The following supporting information can be downloaded at: https://www.mdpi.com/article/10.3390/polym16223219/s1, Figure S1. FTIR spectra of CRA (a) and CRA-brPG (b).; Figure S2. FTIR spectra of Noria (a), Noria-PEEGE (b), and Noria-linPG (c).; Figure S3. UV-vis spectra of (a) CRA in DMSO, containing LiCl and CRA-brPG in water and (b) Noria, PEEGE, and Noria-PEEGE in chloroform.; Figure S4. MALDI-TOF spectrum of CRA-brPG.; Figure S5. MALDI-TOF spectrum of Noria-PEEGE.; Figure S6. Induction of apoptosis after 72 h treatment with Noria-linPG at a concentration of 98 µg/mL of HaCat (left), HFF (middle), HepG2 (right).
