# Supplementary material for: Water-Soluble Polyglycidol-Grafted Ladder Calix Resorcinarene Oligomers with Open Chain and Cyclic Topologies: Synthesis, Characteristics, and Biological Evaluation
Source: Polymers (Basel). 2024 Nov 20;16(22):3219. doi: 10.3390/polym16223219 (PMC11598056; doi:10.3390/polym16223219)
Supplement: Supplementary file 1 [file polymers-16-03219-s001.zip › polymers-3305619-supplementary.pdf]

Supplementary Materials

Water-Soluble Polyglycidol-Grafted Ladder Calix Resorcinarene Oligomers with Open Chain and Cyclic Topologies: Synthesis, Characteristics, and Biological Evaluation

Hristo Penchev [1,*], Erik Dimitrov [1], Christo Novakov [1,2], Emi Haladjova [1,2], Ralitsa Veleva [2,3], Veselina Moskova-Doumanova [2,3], Tanya Topouzova-Hristova [2,3] and Stanislav Rangelov [1,2,*]

1 Institute of Polymers, Bulgarian Academy of Sciences, Akad. G. Bonchev St., Block 103A, 1113 Sofia, Bulgaria
2 Centre of Competence "Sustainable Utilization of Bio-Resources and Waste of Medicinal and Aromatic Plants for Innovative Bioactive Products" (CoC BioResources), 1000 Sofia, Bulgaria
3 Department of Cellular and Developmental Biology, Faculty of Biology, Sofia University St Kliment Ohridski, 8 Dragan Tzankov Blv, 1164 Sofia, Bulgaria
* Correspondence: h_penchev@polymer.bas.bg (H.P.); rangelov@polymer.bas.bg (S.R.)

Synthesis of 3-methoxyphenol

Following the synthetic protocol [1], 55 g (0.5 mol, 1 eq) of resorcinol was dissolved in a 10 % aqueous solution containing 25 g (0.625 mol, 1.25 eq) of sodium hydroxide. 63 g (47.4 ml, 0.5 mol, 1 eq) of dimethyl sulfate were added at vigorous stirring in such a way that the temperature always remained below 40° C. Then the mixture was heated to 100° C for 30 minutes to complete the reaction and to destroy any remaining dimethyl sulfate. After cooling, the organic layer was separated off and the aqueous solution was extracted several times with diethyl ether. The combined organic phases were washed consequently with saturated $NaHCO_3$ solution, water and saturated NaCl solution, after which were dried with anhydrous Na_2SO_4, filtered and fractionated. The fraction, boiling between 100 and 130° C, was collected, and, to remove any dimethylated product, was dissolved in 10% sodium hydroxide solution and extracted several times with diethyl ether. After that, the water layer was acidified and extracted with diethyl ether again to collect the methoxyresorcinol. The ether layer was dried with anhydrous Na_2SO_4, filtered and the ether evaporated on rotary evaporator. The residue was distilled under reduced

pressure giving pure 3-methoxyphenol. Yield 25 g, 39.6%, bp 100-101°C, 2 mbar. ^{1}H NMR (400 MHz, $CDCl_3$) δ 7.14 (t, J = 8.0 Hz, 1H), 6.50 (d, J = 8.3 Hz, 1H), 6.43 (d, J = 8.1 Hz, 2H), 5.48-4.54 (s, 1H), 3.78 (s, 1H).

Synthesis of ethoxyethyl glycidyl ether (EEGE)

EEGE was synthesized following a procedure described elsewhere [2]. p-Toluenesulfonic acid (1 g, 5.80 mmol) was added portion-wise to a magnetically stirred solution of 2,3-epoxypropanol (40.0 g, 0.54 mol) in ethyl vinyl ether (200 mL) maintaining the temperature below 40 °C. The reaction mixture was stirred for 3 h, and then saturated aq. $NaHCO_3$ (100 mL) was added. The organic layer was separated and dried over anhydrous Na_2SO_4, and the ethyl vinyl ether was evaporated under reduced pressure. EEGE was collected by fractional vacuum distillation at 40 °C and 2 mbar as a colorless liquid; a fraction with purity exceeding 98.1% (GC) was used. IR, ATR, cm^{-1} 2979, 2899, 1384, 1338, 1130, 1084, 1050, 856, 533, ^{1}H NMR (600 MHz, $CDCl_3$): δ 4.76 (quint, 1H, J = 5.5 Hz), 3.72-3.38 (m, 4H), 3.15 (m, 1H), 2.80 (m, 1H), 2.62 (dq, 1H, J_1 = 22.1 Hz, J_2= 2.8 Hz) 1.32 (q, 3H, J = 5.5 Hz), 1.20 (td, 3H, J_1 = 7.2 Hz, J_2= 2.0 Hz).

Fourier-Transform Infrared Spectroscopy (FTIR). FTIR spectra were measured with an attenuated total reflection (ATR) spectrophotometer (IRAffinity-1, Shimadzu, Japan) in the 450−4500 cm^{-1} range at a resolution of 1 cm^{-1}.

FTIR spectra of CRA ladder oligomer and the polyglycidol-grafted oligomer, CRA-brPG, are shown in Figure S1. The spectrum of CRA (Figure S1a) has absorption bands at 3335 cm^{-1} resorcinolic O-H stretching, 2875 cm^{-1} C-H stretching, and 1613 cm^{-1} C=C stretching vibrations. The spectrum of CRA-brPG (Figure S1b) exhibits typical bands originating from CRA ladder oligomer – 3335 cm^{-1} O-H stretching, 2875 cm^{-1} C-H stretching, 1651 cm^{-1} C=C stretching, 1138 cm^{-1} C-O stretching vibrations. The intensive new peak at 1038 cm^{-1}, assigned to C-O stretching vibrations, is attributed to polyglycidol moieties.

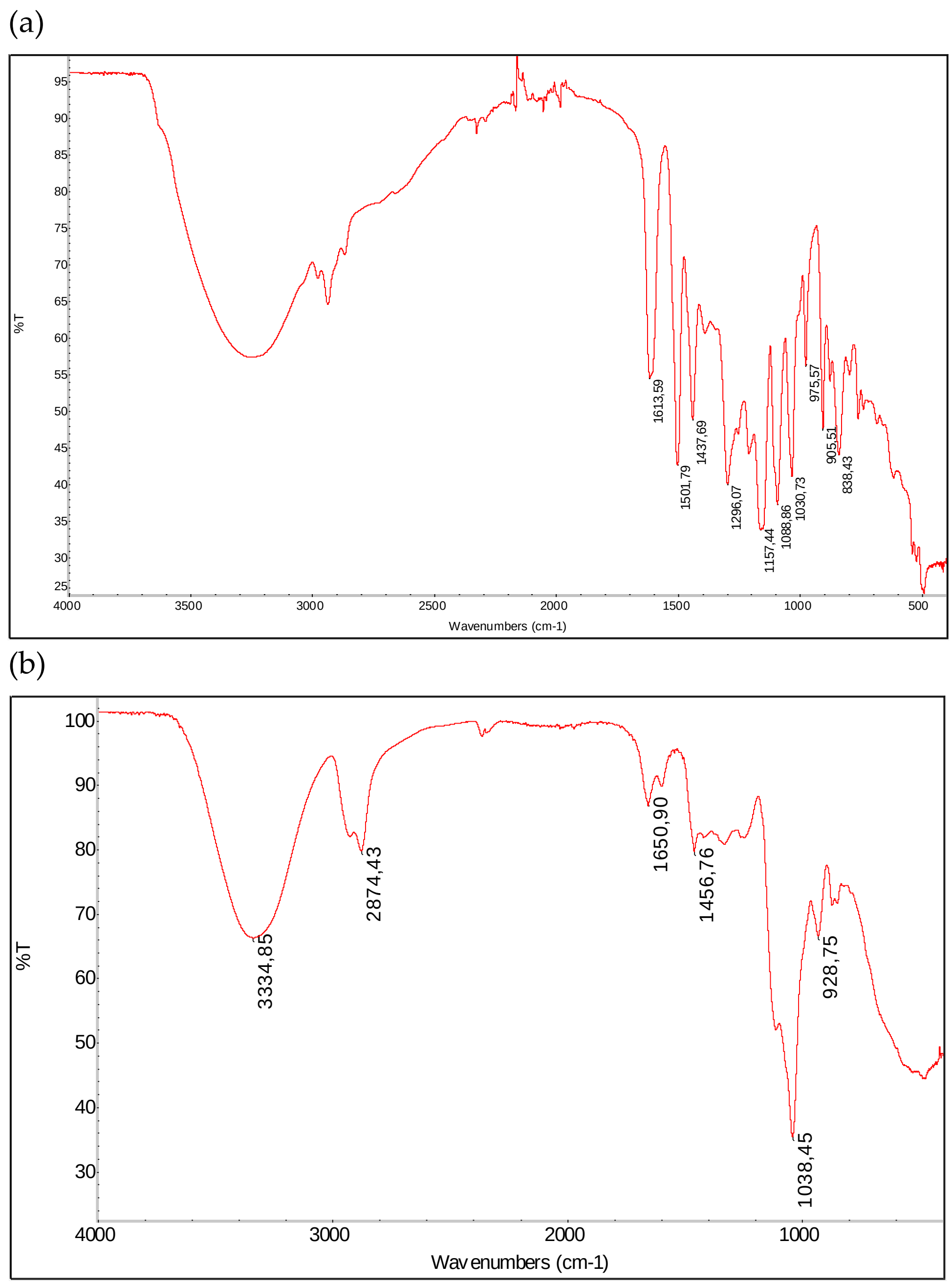

Figure S1. FTIR spectra of CRA (**a**) and CRA-brPG (**b**).

FTIR spectra of the starting Noria ladder oligomer, intermediate Noria-PEEGE, and the final Noria-linPG are shown in Figure S2. The spectrum of Noria (Figure S2a) has absorption bands at 3335 cm^{-1} phenolic O-H stretching, 2931 cm^{-1} C-H stretching, 1606 cm^{-}

^{1} C=C stretching, 1196 cm^{-1} and 1138 cm^{-1} C-O stretching vibrations. The spectrum of Noria-PEEGE (Figure S2b) exhibits bands at 2875 cm^{-1} C-H stretching and 1159 cm^{-1} C-O stretching vibrations, whereas the absorption bands in the spectrum of Noria-linPG (Figure S2c) are 3355 cm^{-1} O-H stretching, 2875 cm^{-1} C-H stretching, 1650 cm^{-1} C=C stretching, 1126 cm^{-1} C-O stretching vibrations.

(a)

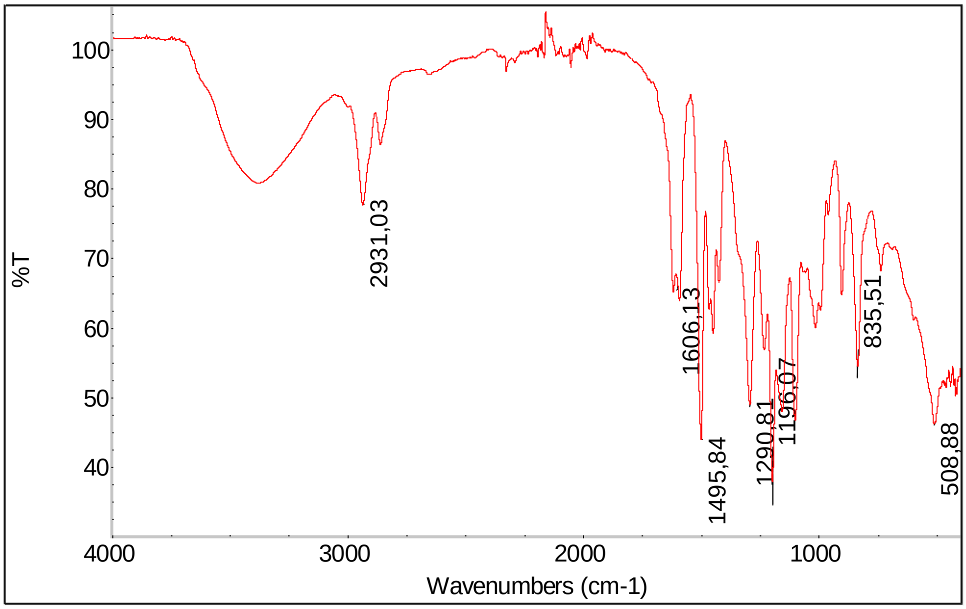

(b)

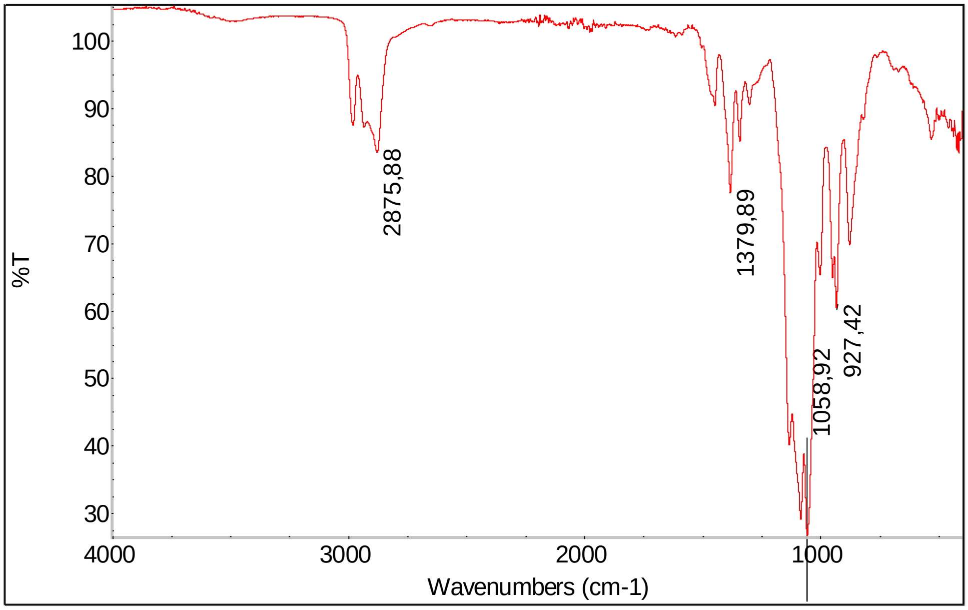

(c)

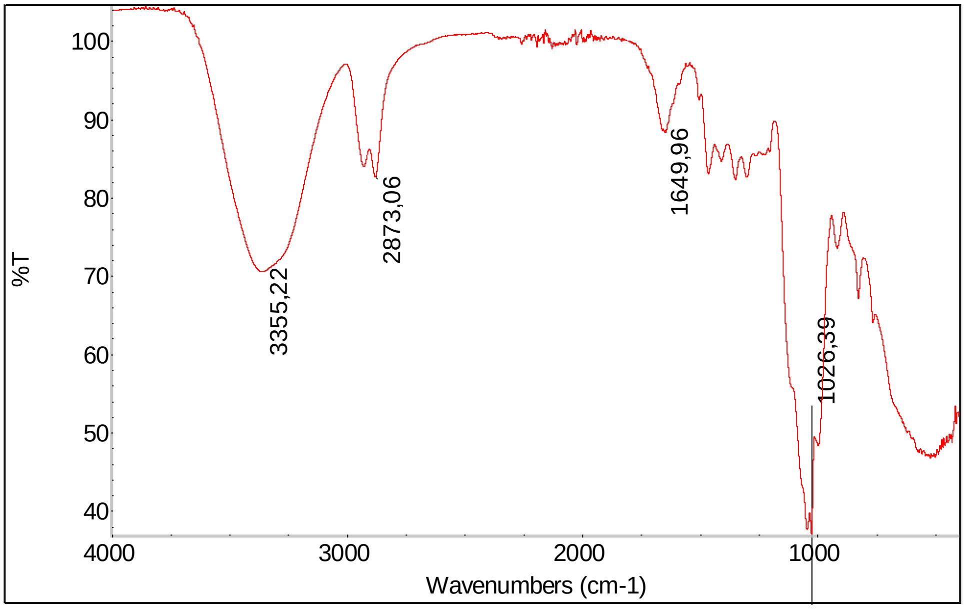

Figure S2. FTIR spectra of Noria (**a**), Noria-PEEGE (**b**), and Noria-linPG (**c**).

UV-Visible spectroscopy. A UV-Vis spectrophotometer (Thermo Scientific, Waltham, MA, USA) was used to record all UV-Vis spectra. All samples, prepared as solutions in DMSO containing LiCl, water or $CHCl_3$ at concentration in the range 0.5 – 1.0 mg/ml, were put in quartz cells with a path length of 1 cm.

UV-vis absorption spectra of CRA and CRA-brPG in DMSO and in water, respectively, are shown in Figure S3a. The CRA oligomer exhibits distinguished benzenoid absorbances at 285, 255 and 218 nm. In the spectrum of CRA-brPG, the peak intensity at 285 nm characteristic for unsubstituted phenolic groups diminishes in intensity and the peak at 255 nm completely disappears.

UV-vis spectra of Noria, PEEGE, and Noria-PEEGE are shown in Figure S3b. The absorption spectrum of pristine Noria exhibits a peak at 245 nm which is attributed to the π-$\pi*$ transition, denoting the electronic transition in cyclic resorcinarene structure due to

electron's elevation from the π bonding orbital to the π∗ antibonding orbital. In the UV-vis of Noria-PEEGE this absorption peak is shifted to 242 nm.

(a) (b)

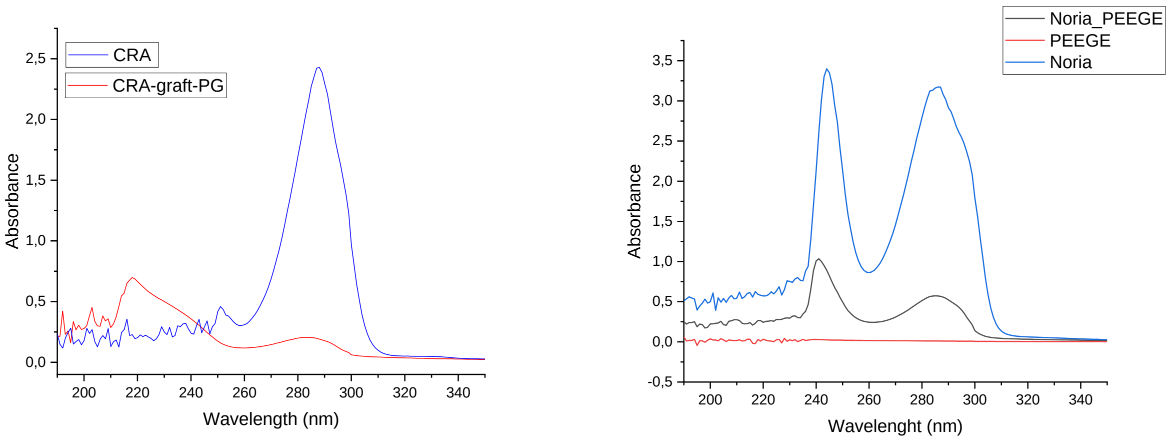

Figure S3. UV-vis spectra of (**a**) CRA in DMSO, containing LiCl and CRA-brPG in water and (**b**) Noria, PEEGE, and Noria-PEEGE in chloroform.

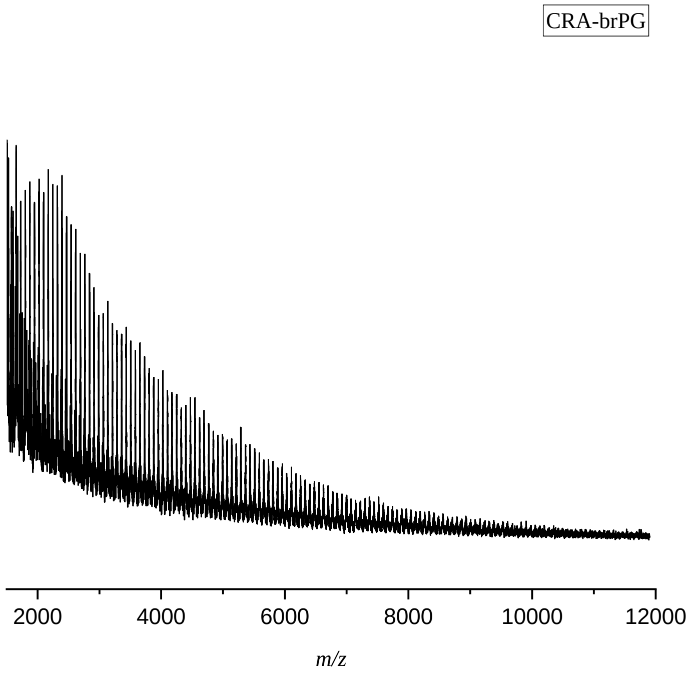

Figure S4. MALDI TOF spectrum of CRA-brPG.

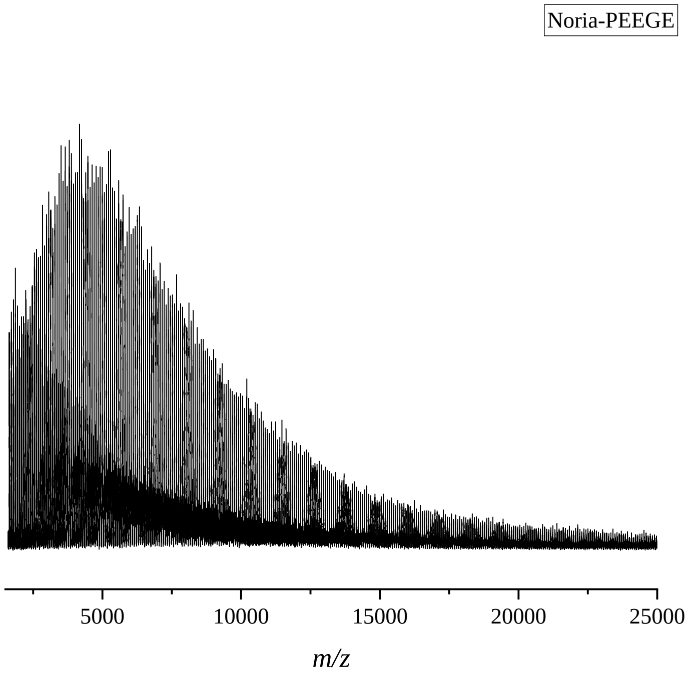

Figure S5. MALDI TOF spectrum of Noria-PEEGE.

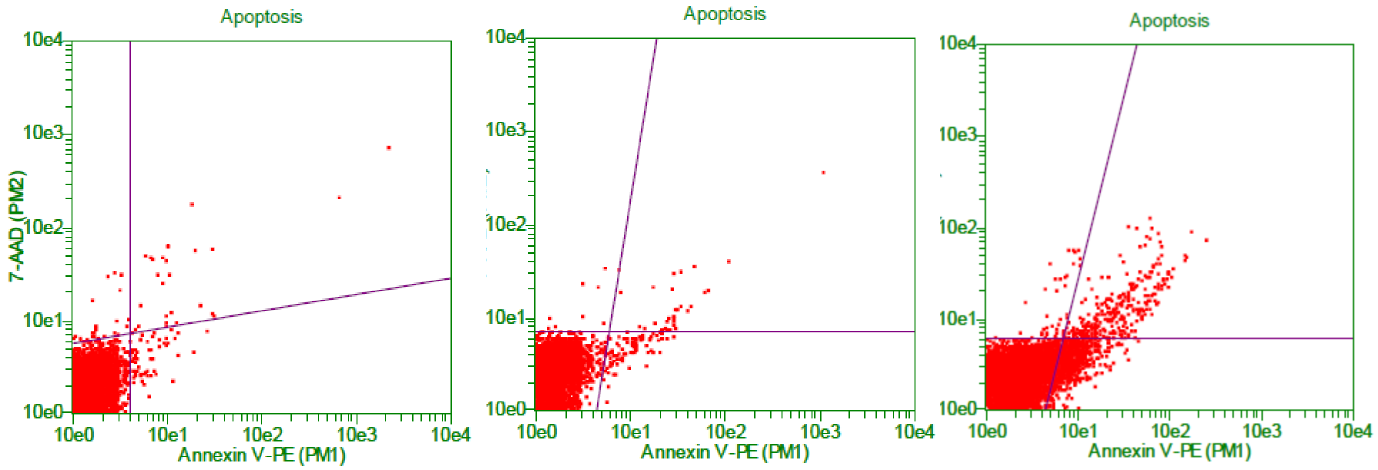

Figure S6. Induction of apoptosis after 72 hours treatment with Noria-linPG at a concentration of 98 µg/ml of HaCat (**left**), HFF (**middle**), HepG2 (**right**).

References

1. Becker, H.; Berger, W.; Domschke, G. Organicum. Practical Handbook of Organic Chemistry, by, Addison-Wesley Pub. Co, 206-207, (1973).
2. Fitton, A.O.; Hill, J.; Jane, D.E.; Millar, R. Synthesis of simple oxetanes carrying reactive 2-substituents. *Synthesis* **1987**, *12*, 1140–1142.
